# Genotyping by Sequencing for SNP-Based Linkage Analysis and the Development of KASPar Markers for Male Sterility and Polyembryony in Citrus

**DOI:** 10.3390/plants12071567

**Published:** 2023-04-06

**Authors:** Rafael Montalt, José Cuenca, María Carmen Vives, Pierre Mournet, Luis Navarro, Patrick Ollitrault, Pablo Aleza

**Affiliations:** 1 Centro de Citricultura y Producción Vegetal, Instituto Valenciano de Investigaciones Agrarias (IVIA), 46113 Valencia, Spain; 2 Agrupación de Viveristas de Agrios (AVASA), 12570 Castellón, Spain; 3 Centro de Protección Vegetal y Biotecnología, Instituto Valenciano de Investigaciones Agrarias (IVIA), 46113 Valencia, Spain; 4 UMR AGAP, CIRAD, 34398 Montpellier, France; 5 UMR AGAP, Institut Agro, CIRAD, INRAE, Université Montpellier, 34060 Montpellier, France

**Keywords:** mandarin, pollen grain, apomixis, association study, marker-assisted selection

## Abstract

Polyembryony and male sterility (MS) are essential characters for citrus breeding. MS, coupled with parthenocarpy, allows for addressing the diversification of diploid seedless mandarin varieties, and nucleocytoplasmic MS is the most prevalent system. Polyembryony limits the use of seed parents in scion breeding programs, and the recovery of monoembryonic hybrids to be used as female parents is a crucial pre-breeding component. The objectives of this work were the identification of SNPs closely linked with the genes implied in these traits for marker-assisted selection. Genotyping by sequencing was used to genotype 61 diploid hybrids from an F1 progeny recovered from crossing ‘Kiyomi’ and ‘Murcott’ tangors. A total of 6444 segregating markers were identified and used to establish the two parental genetic maps. They consisted of 1374 and 697 markers encompassing 1416.287 and 1339.735 cM for ‘Kiyomi’ and ‘Murcott’, respectively. Phenotyping for MS and polyembryony was performed. The genotype–trait association study identified a genomic region on LG8 which was significantly associated with MS, and a genomic region on LG1 which was significantly associated with polyembryony. Annotation of the identified region for MS revealed 19 candidate genes. One SNP KASPar marker was developed and fully validated for each trait.

## 1. Introduction

Among the different traits that characterize the complexity of reproductive biology in plants, male sterility and polyembryony are found within the *Citrus* genera and represent important features of citrus breeding programs.

Male sterility is an important trait because, when coupled with parthenocarpy [1], it allows to partially address the diversification of seedless mandarin varieties at the diploid level. Male sterility has been reported in *Citrus aurantifolia* [2], *C. limon* hybrids [3], *C. medica* [4], *C. sinensis* [2,5], *C. yatsushiro* [2], *C. unshiu* [5], and its hybrids [6]. Several levels and mechanisms of male sterility have been identified in citrus. Chromosomal aberrations, such as asynapsis, reciprocal translocation, and failure of spindle formation, are important phenomena causing pollen sterility. For example, reciprocal translocation is found to cause pollen sterility in the ‘Valencia’ sweet orange (*C. sinensis*) [6], inversion is the cause of partial pollen sterility in the ‘Mexican’ lime (*C. aurantifolia*) [6], and asynapsis with a genetic determinant has been identified in the ‘Mukaku Yuzu’ (*C. junos*), while this is induced by low temperature in the ‘Eureka’ lemon (*C. limon*) and the ‘Mexican’ lime [6,7]. Besides chromosomal aberration, nucleocytoplasmic male sterility (CMS) is the most prevalent system in citrus, and it has been proposed that satsuma (*C. unshiu*) and progenies derived from satsuma (as female parent) display CMS caused by the cooperative action of both cytoplasmic and nuclear genes. Several studies have been performed to decipher the genetic control of male sterility derived from satsuma. Yamamoto et al. [8] demonstrate the interaction between nuclear and cytoplasmic genes by reciprocal hybridizations. Subsequent research has pointed to the involvement of nuclear genes in male sterility [9,10,11,12,13,14,15]. DNA marker analysis for nuclear and cytoplasmic genomes and genome-wide SNP marker analysis showed that CMS in the satsuma was derived from its seed parent, the ‘Kishu’ mandarin (*C. kinokuni*), and that the nuclear genes come from the male parent, ‘Kunenbo’ mandarin (*C. nobilis*) [15,16,17]. Goto et al. [14] posited that male sterility was associated with failed pollen grain development and scant viability. These authors compute the index of male sterility in a population derived from satsuma using two parameters: (i) the number of pollen grains per anther (NPGA), and (ii) the apparent pollen fertility (APF). Both parameters are inherited by their progeny, suggesting the involvement of a nuclear factor. Recently, two QTLs related to male sterility have been reported: MS-P1, which is a major QTL for reducing the number of pollen grains per anther; and MS-F1, related to lower apparent pollen fertility [15]. However, the resolution of the genetic map was too low to develop efficient markers for early selection. For instance, one objective of this work is the development of new markers associated with male sterility trait for seedless breeding.

Apomixis (asexual embryo formation) has been observed in more than 400 plant species [18]; however, apomixis is not particularly common in agriculturally important woody crops, with the exception of apple, mango, and citrus [19,20]. In citrus, apomixis is sporophytic (also referred to as adventitious embryony from nucellar cells) [3,19], and it is present in most genotypes, with the exception of citron, pummelo, clementines, and some mandarin hybrids. The seeds of non-apomictic genotypes, also called monoembryonic genotypes, contain only one sexual embryo, whereas in apomictic genotypes (polyembryonic), there is one sexual embryo and multiple nucellar embryos genetically identical to the mother plant. In the seeds of polyembryonic citrus genotypes, the formation of the nucellar embryos can be initiated before fertilization [21], and competition between the zygotic and nucellar embryos generally results in the failure of the development of the zygotic embryo [3,19]. This characteristic is a strong limitation for using polyembryonic genotypes as female parents in sexual hybridizations, since it hampers the recovery of large hybrid populations. In programs aiming to introgress specific traits over several cycles of hybridization, the recovery and selection of monoembryonic hybrids to be used as female parents for further breeding is crucial due to the low number of parents available. Considering the very long juvenile phase in citrus, the development of molecular markers for marker-assisted selection (MAS) appears particularly important for this trait. At the opposite end of the scale, polyembryony is very advantageous for rootstock production, since plants obtained from polyembryonic seeds are identical to the mother plant. That is why rootstock breeding programs look for polyembryonic hybrids to ensure clonal propagation by seedlings of the newly selected rootstock. Therefore, the development of markers associated with monoembryony and polyembryony will be very useful for MAS in both varieties and rootstock breeding programs.

On the basis of genomic analyses of primitive, wild, and cultivated citrus, Wang et al. [22] highlighted the emergence of apomixis during citrus domestication. These authors narrowed down the genetic locus responsible for citrus polyembryony to an 80 kb region located on chromosome 4 of the Chinese pummelo genome assembly (MKYQ00000000.1), containing 11 candidate genes. Among these genes, a candidate gene, *CitRWP,* was identified for the single dominant allele responsible for polyembryony, and a miniature inverted-repeat TE (MITE) insertion in the promoter region of the *CitRWP* gene cosegregated with the polyembryonic phenotype [22]. Later, Shimada et al. [23] reported the candidate gene, *CitRKD1*, at the polyembryonic locus, which plays a principal role in regulating somatic embryogenesis. These authors suggested that a MITE insertion in the upstream region might be involved in regulating the *CitRKD1* transcription. For instance, the *CitRKD1* gene comprises two alleles, polyembryonic allele with a MITE insertion, and monoembryonic without a MITE insertion. Recently, Catalano et al. [24] confirmed the allelic configuration for *CitRKD1* in different lemon genotypes using MITE primers. Alongside that, based on this MITE insertion, our research group developed an InDel marker, which has been evaluated in segregation progenies and germplasm genetic diversity, obtaining good results with the genotypes analyzed. However, InDel analysis is time-consuming (PCR products must be resolved by electrophoresis in long-agarose gel), as well as expensive for studying a large number of progenies. Therefore, it is important to develop alternatives, such as SNP (Single nucleotide polymorphism) markers based on KASPar technology, to simplify and make the analysis faster and cheaper.

SNP genotyping by the KBiosciences Competitive Allele Specific PCR SNP genotyping (KASPar) technology is simple and cost-effective for genotyping a limited number of markers in large populations, as compared with other SNP genotyping assays. Highly efficient protocols have been adapted to work with citrus by Cuenca et al. [25] and Garcia-Lor et al. [26]. It therefore appears to be a very well-adapted methodology for MAS. The efficiency of MAS is directly linked to the vicinity of the used markers with the genes or factors directly implied in the expression of the targeted trait. The ability to identify candidate genes associated with useful traits has progressed significantly with the development of next-generation sequencing (NGS) technologies, thereby facilitating the massive identification of SNP markers in large populations, as well as working on reduced genome representations. Examples include restriction-site-associated DNA sequencing (RADseq) [27,28], diversity array technology sequencing (DArTseq) [29], and genotyping by sequencing (GBS) [30,31]. In citrus GBS, RAD sequencing and DARTSeq have been successfully developed and used to study germplasm diversity and decipher related phylogenomic structures [32,33,34], high density genome mapping [35,36,37,38], as well as QTL analyses [39] and genome-wide association studies (GWAS) [40].

The aims of this study were: (i) the identification of SNPs closely linked with male sterility and polyembryony. This was carried out by combining GBS data and analyzing the number of pollen grains per anther (NPG), apparent pollen fertility (APF), and polyembryony in a segregant progeny recovered from a cross between the male sterile and monoembryonic ‘Kiyomi’ tangor (*C. unshiu* × *C. sinensis*) as the female parent, and the male fertile and polyembryonic ‘Murcott’ tangor (*C. sinensis* × unknown mandarin) as the male parent); (ii) the identification of candidate genes associated with male sterility; and (iii) the development and assessment of SNP markers based on KASPar technology for MAS in citrus breeding programs for these two important traits.

## 2. Results and Discussion

### 2.1. SNP Calling

According to the initial parameters indicated in the Section 3, the TASSEL software identified 22,326 di-allelic SNPs. We then filtered the positions where all replicates of the parents were identical, with at least one of the parents being heterozygous; other filters included those with less than 15% missing data, and with at least 10% minor allele frequency. This resulted in the selection of 6444 SNPs.

### 2.2. Genetic Linkage Maps of ‘Kiyomi’ and ‘Murcott’ Tangors: Synteny and Collinearity with the Reference Genome of Clementine

The SNP matrix, containing 6444 segregating markers and 61 individuals, was used to construct the genetic maps of the ‘Kiyomi’ and ‘Murcott’ tangors. Markers which were heterozygous for the ‘Kiyomi’ tangor and homozygous for the ‘Murcott’ tangor were filtered for the linkage mapping of the ‘Kiyomi’ tangor. Markers which were heterozygous for the ‘Murcott’ tangor and homozygous for the ‘Kiyomi’ tangor were filtered for the linkage mapping of the ‘Murcott’ tangor. Markers with unexpected segregation according to the parents were eliminated. By the end of this process, in order to optimize the quality of genotyping data, only the SNPs within genes were selected, and only one marker per gene was conserved to limit the redundancy of the markers. The number of discarded markers in these filters is provided in Supplementary Table S1.

Linkage mapping of the ‘Kiyomi’ tangor was performed using a matrix of 1396 segregating SNPs and 61 individuals. A total of 1374 SNPs were assigned to one of the nine resulting linkage groups (LGs), which corresponds to the number of haploid chromosomes in citrus (Table 1 and Supplementary Table S2). The number of markers was unequally distributed among the LGs. LG6 included only 53 SNPs, while 324 SNPs were attributed to LG3. The small number of markers found in LG6 was due to the high homozygosity of the ‘Kiyomi’ tangor in a large part of the corresponding chromosome. LG1 displayed the lowest genetic size (96.8 cM). LG3, comprising 324 SNPs, displayed the largest genetic size (237.7 cM) (Table 1). The entire map spanned 1416.3 cM, with an average interlocus distance of 1.04 cM. A total of 87% of SNPs had an interlocus gap of less than 3 cM, 12.6% of SNPs had an interlocus gap between 3 and 10 cM, and only 0.4% had a gap measuring more than 10 cM. Most of the LGs were composed of SNPs mapped onto the syntenic pseudo-chromosomes (Sc) of the clementine reference genome. The Circos representation and the Marey map plot between the genetic and physical locations over the clementine reference genome are provided in Supplementary Figure S1. The genetic map displayed high global synteny (98%). LG1, LG4, and LG9 displayed full synteny with the reference genome. LG2 (one in Sc5), LG3 (one in Sc1 and one in Sc2), LG5 (one in Sc4), and LG6 (one in Sc3 and one in Sc8) displayed almost full synteny. LG7 and LG8 stood out. Two, one, and six markers physically located on chromosomes 1, 4, and 5, respectively, were genetically mapped on LG7. One, six, and five markers physically located on chromosomes 1, 3, and 9, respectively, were mapped on LG8.

Linkage mapping of the ‘Murcott’ tangor was performed using a matrix of 737 segregating SNP markers and 61 individuals. A total of 697 were assigned to one of the nine resulting LGs, and the number of markers ranged from 40 for LG2 to 168 for LG8 (Table 1 and Supplementary Table S3). The total size of the genetic map was 1339.7 cM, with an average interlocus distance of 1.95 cM. The smallest LG was LG9, measuring 103.4 cM, while LG3 was the largest (231.3 cM). The interlocus gap of 74.9% of the SNPs was less than 3 cM, 23.3% of SNPs had an interlocus gap between 3 and 10 cM, while the genetic distance was more than 10 cM in only 1.9% of SNPs. Overall, synteny was high (96.1%) between the ‘Murcott’ tangor genetic map and the clementine reference genome (Supplementary Figure S2). LG2, LG5, and LG9 displayed full synteny with the reference genome. LG1 (one in Sc6), LG3 (one in Sc4, one in Sc8, and one in Sc9), LG4 (one in Sc2 and one in Sc3), LG6 (one in Sc3 and two in Sc8), and LG7 (one in Sc4 and two in Sc5) displayed almost full synteny. As already observed in the ‘Kiyomi’ tangor, LG8 had more SNPs that were not mapped on the corresponding pseudo-chromosome, with counts of four and 11 SNPs (out of a total of 168) located on the physical assembly of pseudo-chromosomes 3 and 9, respectively.

The ‘Kiyomi’ genetic map displayed high collinearity with the clementine reference genome, although incongruency between the genetic map and the physical positions over the reference genome was observed in a cluster of 14 markers between 30 and 34 cM on LG3. This misplaced genomic region was also shown for ‘Murcott’. However, only two markers were concerned due to the low number of heterozygous markers in this genomic region for ‘Murcott’. Additional shared discrepancies between the ‘Kiyomi’ and ‘Murcott’ genetic maps, and the *C. clementina* v1.0 assembly were observed for markers of chromosomes 3, 5 and 9 located, respectively, on LGs 8, 7, and 9. Similar discrepancies for the same genomic regions of the *C. clementina* v1.0 assembly were also identified in the high-density genetic maps of sweet orange and trifoliate orange [37], as well as in the reference genetic map of clementine [41] and ‘Fortune’ (*C. clementina* × *C. tangerina*) and ‘Ellendale’ (*C. reticulata* × *C. sinensis*) [38]. In this regard, Ollitrault et al. [38] suggested that most of the apparent non-syntenic or non-colinear markers were rather due to minor errors in the clementine genome assembly. Overall, the high synteny and collinearity with the clementine reference genome shown in the two genetic maps is consistent with previous studies concluding high synteny and collinearity between *Citrus* species [38,41,42,43]. ‘Murcott’ and ‘Kiyomi’ tangors are interesting parents, widely used for mandarin breeding, and the high-density genetic maps presented here can prove useful for optimizing their use in breeding programs.

### 2.3. Phenotypes and Marker–Trait Association Studies

#### 2.3.1. Phenotypes

Among the 61 genotyped hybrids, 53 flowered during the three-year experiment, 52 of them produced fruits, and in 32 of these, the fruits contained seeds. Data obtained for the phenotyped traits NPGA and APF, as well as the polyembryony of each hybrid, are displayed in Supplementary Table S4.

Male sterility phenotyping was performed based on NPGA and APF. The ANOVA analysis showed significant differences between genotypes for NPGA, while no differences were observed between genotypes for APF (Table 2).

**Figure 1:**
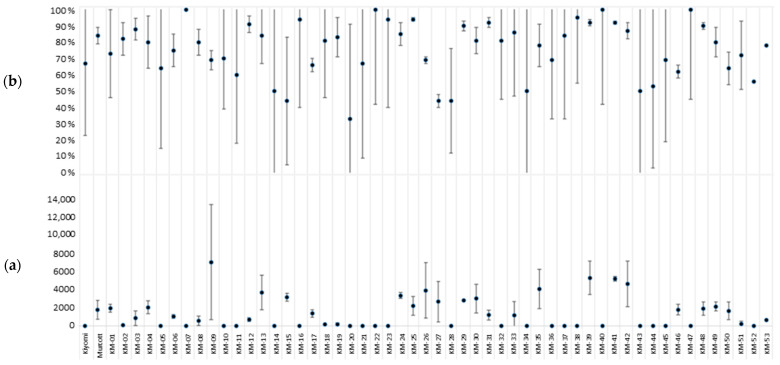
Average and standard deviation representation of (**a**) the number of pollen grains per anther and (**b**) the percentage of apparent pollen fertility in the ‘Kiyomi’ × ’Murcott‘ offspring phenotyped for male sterility. The *x*-axis indicates the name of the parents and hybrids.

As shown in Figure 1, great differences, particularly for APF, were observed within several genotypes, while the data obtained for NPGA were more homogeneous within genotypes. Taking both parameters together, some genotypes showed high APF, but very low NPGA values. For example, for KM-1 and KM-2 with similar APF averages of 73% and 82%, respectively, the average NPGA values were 1950 and 81, respectively. Therefore, KM-1 is a male fertile hybrid, while KM-2 is practically a male sterile hybrid (Figure 1 and Supplementary Table S4).

None of the hybrids with high NPGA values showed APF values low enough to cause male sterility, suggesting that NPGA is the key factor in male sterility in the ’Kiyomi‘ × ‘Murcott’ offspring. In this line, Goto et al. [14] evaluated NPGA and APF in a satsuma progeny, and reported that male sterility is primarily caused by decreased NPGA. Although satsuma is generally described as male sterile, several studies have pointed out that male sterility in satsuma is partial and influenced by both environmental conditions and genotype. In fact, new varieties have been obtained using pollen from satsuma [44]. In this regard, Yang and Nakagawa [45,46] reported that temperature treatments at 15 °C and 20 °C during flower bud growth and development are favorable in the recovery of male fertility in satsuma. In addition to this, a low degree of male fertility has also been achieved under field conditions, as has been shown by Vithanage [47], who reported two seeds per fruit when ‘Ellendale’ tangor was pollinated with satsuma, and by Goto et al. [14], who reported an average of 389 NPGA in ‘Okitsu wase’ satsuma. In the same paper, Goto et al. [14] reported that pollen grains in ‘Kiyomi’ (satsuma × sweet orange) were not detected, suggesting that male sterility in ‘Kiyomi’ is stricter than in satsuma. In this study, we have observed an average of one NPGA in ‘Kiyomi’. This very low value points to strict male sterility in ‘Kiyomi’, and the fact that it is not complete. Beyond the differences in NPGA values between hybrids producing low numbers of pollen grains, Goto et al. [15] suggested that the release of pollen grains from anthers occurs when a certain NPGA value is exceeded. They also assumed that the presence of less than approximately 1300 NPGA was a crucial criterion for male sterility. In this study, we have observed pollen grain release in those anthers with more than 1000 NPGA. Thus, we have established 1000 NPGA as the criterion of male sterility.

**Figure 2:**
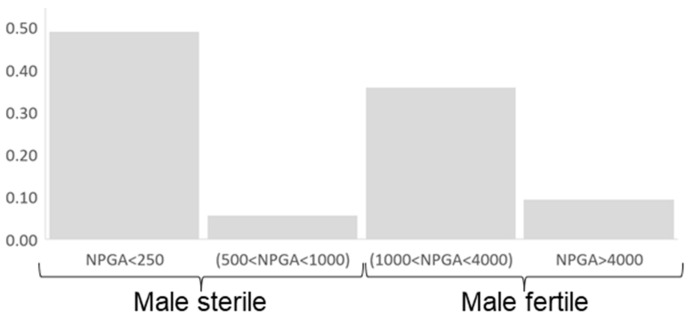
Histogram displaying the number of pollen grains per anther (NPGA) distribution in the diploid hybrids analyzed.

In Figure 2, we display the histogram obtained for NPGA. A total of 49% of the hybrids plus ‘Kiyomi’ produced less than 250 NPGA, and 6% produced between 500 and 1000 NPGA; 36% of the hybrids plus ‘Murcott’ produced between 1000 and 4000 NPGA, and 9% produced more than 4000 NPGA (Figure 2 and Supplementary Table S4).

As expected, all seeds of the ‘Kiyomi’ tangor were monoembryonic, and all seeds of the ‘Murcott’ tangor were polyembryonic. Of the 32 hybrids that produced fruits with seeds, 12 of them produced monoembryonic seeds (37.5%), and the other 20 (62.5%) hybrids produced polyembryonic seeds. In the last group, we found nine hybrids with solely polyembryonic seeds, and 11 hybrids with percentages of polyembryonic seeds ranging between 14 and 92% (Supplementary Table S4).

#### 2.3.2. Marker–Trait Association

Through a general linear model (GLM) using default parameters in the TASSEL 5 software, polyembryony and male sterility marker–trait association studies were separately evaluated in both ‘Kiyomi’ and ‘Murcott’ tangors maps. The statistical significance of the genetic and phenotypic associations was calculated with a 0.05 probability threshold, as well as applying the Bonferroni correction for multiple testing. For the `Murcott’ tangor, the probability threshold was *p* ≤ 7.5 × 10^−5^ (0.05/670) or −log(*p*) >= 4.01, whereas for `Kiyomi´, it was *p* ≤ 3.7 × 10^−5^ (0.05/1346) or −log(*p*) >= 4.43.

The TASSEL software was designed to evaluate trait associations, evolutionary patterns, and linkage disequilibrium using GWAS. It has been successfully used for marker–trait association studies using bi-parental progenies. Applying GLM, Sumitomo et al. [48] tagged SNP markers onto the flower color genes in autohexaploid *Chrysanthemum*, and Shibaya et al. [49] identified QTLs for root color and carotenoid contents in carrot. In Japanese plum, Salazar et al. [50] identified QTLs linked to fruit quality traits using three F1 progenies with a common female parent.

**Figure 3:**
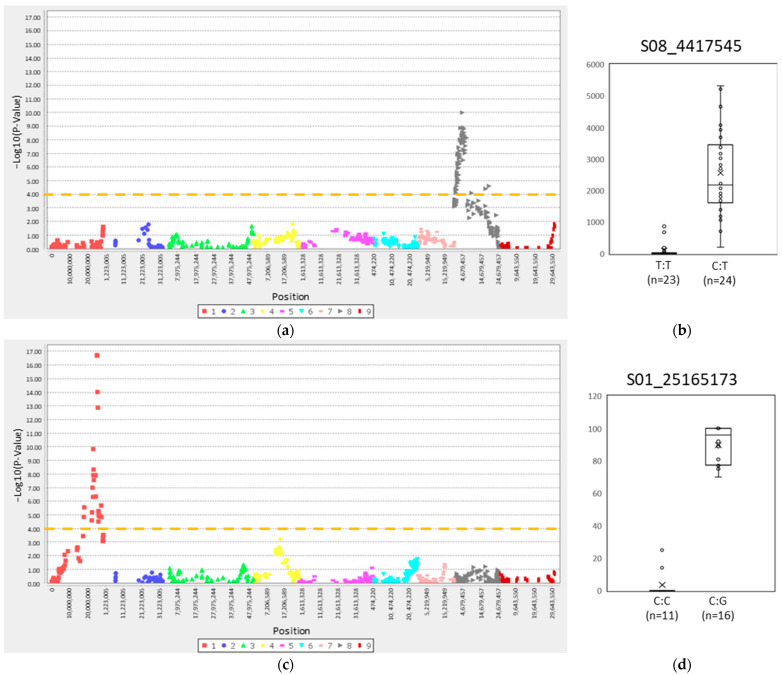
Association study for ‘Murcott’ gametes. (**a**,**b**) Number of pollen grains per anther; (**a**) Manhattan plot. (**b**) Box plot representation for the most significant marker, SNP S08_4417545. (**c**,**d**) Polyembryony. (**c**) Manhattan plot. (**d**) Box plot representation for the most significant marker, SNP S01_25165173. The orange line is the threshold for significant *p*-values.

Here, a genotype–phenotype association study for male sterility was performed for the NPGA trait. APF was not used for association studies, since no significant differences were observed between the hybrids. In the ‘Murcott’ gamete map, the GLM identified 68 SNP markers with statistical significance (Supplementary Table S5). All of them were located on 68 different genes on LG8. A total of 49 of these markers were clustered in a region of 4.79 Mb (between the positions 2107212 and 6899421 bp), corresponding to a genetic region of 26.735 cM (between 26.771 and 53.506 cM) (Figure 3a and Supplementary Table S5). The most significant marker identified was S08_4417545, with a *p*-value of 1.02E-10 (LOD = 9.99) and a genetic position of 46.436 cM (Supplementary Table S5).

All hybrids with the TT allelic configuration for the SNP S08_4417545 were male-sterile. The vast majority of the hybrids with the CT allelic configuration produced more than 1000 NPGA, none of them showing very low values for NPGA (Figure 3b). It should be noted that some significative SNPs were identified outside of the clustered region (between 26.771 and 53.506 cM) (Figure 3a and Supplementary Table S5), therefore it is likely that other genomic regions on LG8 can be involved in male sterility. Through QTL mapping of a population derived from two satsuma hybrids, Goto et al. [15] identified three QTLs (*MS-P1*, *MS-P2* and *MS-P3*) associated with NPGA. The most associated *MS-P1*, located on LG8 (genetic position 37.5 cM), may correspond to the association genomic region for NPGA identified in our GLM analysis. In addition, the two other QTLs with lower associations, *MS-P2* and *MS-P3*, both located on LG6b and separated by a genetic distance of 29 cM, suggest that other genomic regions can also be involved in this trait.

For polyembryony, through the genetic association study performed in the ‘Murcott’ gamete map, GLM identified 25 SNP markers with statistical significance, all of them located on 25 different genes on chromosome 1. These markers were clustered in a region of 9.85 Mb (located between positions 17831378 and 27687717 bp), corresponding with a genetic region of 64.127 cM (between 79.356 and 143.483 cM) (Figure 3c and Supplementary Table S5). For the most significant marker (S01_25165173), all hybrids with the CG allele produced polyembryonic seeds, the total of which exceeded 25% of the total (Figure 3d). Averages of polyembryonic seeds between 69.8 and 91.4% have been reported in apomictic genotypes by Kishore et al. [51]. We performed a BLASTn search of the sequence of the *CitRWP* gene of the pummelo genome in the genome assembly of *C. clementina* v1.0 Wang et al. [22] demonstrated that the insertion of a MITE in close vicinity of this gene was responsible for polyembryony in mandarins. The BLAST analysis identified the annotated gene, Ciclev10010497m, as the homologue of *CitRWP* with a high-scoring segment pair (HSP), with a positive identity of 99.89%. This gene is located on chromosome 1 at position 25480488–25482037 bp of the *C. clementina* assembly. The genomic regions of 25165173–25690547 bp on chromosome 1, defined by the markers included within a 5 cM interval each side of the higher signal marker in our association study, include the Ciclev10010497m location. Therefore, our results are in full agreement with previous conclusions regarding the importance of *CitRWP* for polyembryony in mandarin.

### 2.4. Gene Annotations of the Genomic Region Associated with Male Sterility

The 4.79 Mb genomic region identified in our GWAS was examined for gene annotations in the clementine genome (https://phytozome-next.jgi.doe.gov [52] (accessed on 7 June 2022). A genomic region (between 5913054 and 6901468 bp) containing 67 annotated genes, 19 of which are involved in different biological pathways that may affect pollen formation or development, draws our attention. These genes include papain-like cysteine protease enzymes, pentatricopeptide repeat, ATP binding, plant homeodomain-finger family protein, WD40 repeat-like, and 3-oxo-5-alpha-steroid 4-dehydrogenase. Gene annotations are provided in Supplementary Table S6.

The annotation functions of two genes (Ciclev10028670m.g and Ciclev10029917m.g) are associated with papain-like cysteine protease enzymes. Cysteine protease plays a critical regulatory role in programmed cell death (PCD), and its regulation is influenced by temperature stress [53]. Tapetum, a layer of cells surrounding microspores, is key in pollen development, providing nutritive proteins, enzymes, and sporopollenin precursors for pollen maturation [54]. During the late stages of pollen development, tapetum undergoes PCD, and either premature [55] or inhibition [56] PCD of tapetal cells will result in male sterility. The importance of cysteine proteases in pollen formation has been reported in tobacco [57,58,59], *Arabidopsis* [60,61], soybean [62], tomato [63], rice [64], cabbage [65], and brassica [66].

In total, five genes around SNP08_6142645 (Ciclev10030265m.g, Ciclev10029744m.g, Ciclev10028481m.g, Ciclev10030279m.g, and Ciclev10029914m.g) are annotated as Pentatricopeptide repeat (PPR). The PPR superfamily protein represents the most frequent protein class identified as restorers of fertility (Rf). Examples of PPRs characterized as Rf and confirmed through transgenic analysis include *Rf-PPR592* in petunia, *Rfo* in radish, and *Rf1A*, *Rf1B*, *Rf3*, *Rf4*, *Rf5*, *Rf6*, *GRP162,* and *PPR762* in rice (reviewed in Gaborieau et al. [67]).

Another nine genes around SNP08_6142645 (Ciclev10029967m.g, Ciclev10028124m.g, Ciclev10030242m.g, Ciclev10028233m.g. Ciclev10030082m.g, Ciclev10029947m.g, Ciclev10030361m.g, Ciclev10028181m.g, and Ciclev10030145m.g) are annotated as related to ATP binding, a transport protein involved in sporopollenin (the material that forms the durable, chemically stable outer layer on pollen grains) export and/or shuttling from the tapetum. Chang et al. [68] reported that the *OsABCG3* gene (an ATP binding cassette) is essential for pollen development in rice. Other genes in this genomic region include Ciclev10029260m.g, which encodes a plant homeodomain (PHD)-finger family protein. In *Arabidopsis*, the male meiocyte death1 gene encodes a PHD-finger protein which is required for male meiosis [69]; Ciclev10028263m.g encodes a WD repeat protein, which regulates pollen growth and viability in Flax (*Linum usitatissium* L.) [70]; and Ciclev10028796m.g with the 3-oxo-5-alpha-steroid 4-dehydrogenase domain localized on the C-terminal part of Polyprenol reductase2, of which the deficiency causes male sterility in *Arabidopsis* [71].

Pollen grain number in angiosperms is a key reproductive trait that has been studied extensively for decades. Despite its agricultural and evolutionary importance, the genetic basis of the pollen grain number has remained elusive, primarily due to its quantitative nature [72]. The information generated from gene annotations allows us to focus our efforts on 19 genes related to male sterility over the 67 genes annotated in the genomic region identified by the QTL analysis. This limited number will now allow for the development of affordable, albeit time-consuming, approaches to determine whether these genes are actually involved in the male sterility and citrus interaction. Further experiments will be necessary to shed light on this complex trait of citrus reproductive biology.

### 2.5. Development and Validation of SNP Markers Associated to Male Sterility and Polyembryony

According to our GWAS analysis, we developed one KASPar SNP marker for each trait. For male sterility, the candidate region, between 5,913,054 and 6,901,468 Kb, contained three SNPs: S08_6026790 in Ciclev10027952m.g, S08_6050573 in Ciclev10027768m.g, and S08_6142645 in Ciclev10028670m.g (Supplementary Table S6). Since Ciclev10028670m.g is annotated to encode papain-like cysteine protease enzymes (of which the importance in pollen formation has been widely reported), we chose S08_6142645 (hereinafter SNP8) for the development of the male sterility KASPar SNP marker. SNP8 is located on the physical position 6,142,645 on chromosome 8 of the *C. clementina* v1.0 genome assembly, corresponding to the genetical position 53.506 cM on the ‘Murcott’ map.

In the framework of our breeding program, a progeny of 20 diploid hybrids obtained from open pollinated ‘Kiyomi’ tangor was phenotyped for male sterility and analyzed thorough KASPar with the S08 SNP marker (Table 3).

**Table 3:** Genetic analysis of 20 hybrids recovered with ‘Kiyomi’ as a female parent and an unknown male parent, with the S08_6142645 SNP marker associated to male sterility.

Individual	Phenotype	Genotype
Kiyomi × Unknown-1	Fertile	CT
Kiyomi × Unknown-2	Sterile	CC
Kiyomi × Unknown-3	Sterile	CC
Kiyomi × Unknown-4	Fertile	CT
Kiyomi × Unknown-5	Sterile	CC
Kiyomi × Unknown-6	Fertile	CT
Kiyomi × Unknown-7	Fertile	CT
Kiyomi × Unknown-8	Sterile	CC
Kiyomi × Unknown-9	Fertile	CT
Kiyomi × Unknown-10	Sterile	CC
Kiyomi × Unknown-11	Sterile	CC
Kiyomi × Unknown-12	Fertile	CT
Kiyomi × Unknown-13	Fertile	CT
Kiyomi × Unknown-14	Sterile	CC
Kiyomi × Unknown-15	Fertile	CT
Kiyomi × Unknown-16	Fertile	CT
Kiyomi × Unknown-17	Fertile	CT
Kiyomi × Unknown-18	Fertile	CT
Kiyomi × Unknown-19	Fertile	CT
Kiyomi × Unknown-20	Sterile	CC

**Figure 4:**
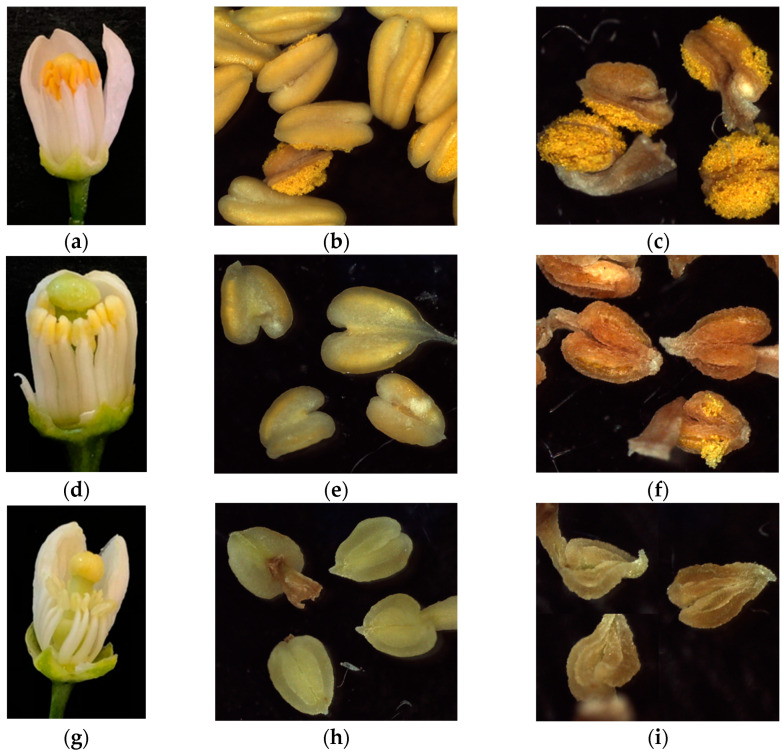
Different types of flower phenotypes observed in the segregation progeny recovered from the cross between ‘Kiyomi’ and ‘Murcott’. (**a**–**c**) High number; (**d**–**f**) moderate-to-low; (**g**–**i**) very low-to-null pollen grain quantity. This quantification was observed in flowers at anthesis (**a**,**d**,**g**), fresh anthers (**b**,**e**,**h**), and dehiscent anthers (**c**,**f**,**i**).

Male fertile or sterile phenotype is based on the observations of both the fresh anthers color and the quantity of pollen grains in dehiscent anthers (see Figure 4). KASPar plot obtained with the S08_6142645 SNP marker is provided in Supplementary Figure S3.

A total of eight of the 20 hybrids produced flowers with pale yellow or off-white anthers and low-to-null pollen quantity. All of these hybrids displayed CC allelic configuration for SNP8. On the other hand, 12 hybrids displayed flowers with a fertile phenotype (yellow anthers and a high quantity of pollen grains) associated with CT allelic configuration for this SNP. In addition, 11 different commercial mandarin cultivars were analyzed (Supplementary Table S7). ‘Okitsu’ satsuma, ‘Kiyomi’ tangor, and ‘Queen’ mandarin were classified as male sterile (CC), whereas the other mandarins were genotyped as fertile, with CT allelic configuration for ‘Nadorcott’, ‘Murcott’, ‘Kara’, and ‘Encore’ mandarins, and TT allelic configuration for ‘Clemenules’, ‘Campeona’, ‘Fortune’, and ‘Ellendale’. It is expected that when crossing a male sterile (CC) cultivar as the seed parent with pollen from a fertile (CT) cultivar, 50% of the hybrids will be male sterile and the other 50% will be fertile, meanwhile 100% of the hybrids will be male fertile when using pollen from a (TT) fertile cultivar. Nevertheless, this SNP marker has been tested in these two populations with very good results and it would be of interest to test in others genetic contexts, that we do not have at this moment.

Regarding polyembryony, among the most significant SNPs identified in our association study for polyembryony, S01_25497528 was the most closely positioned to the blasted sequence of the *CitRWP* gene [22] in the *C. clementina* v1.0 reference. Thus, we used this SNP to develop the hereinafter SNP1, located on chromosome 1 of the *C. clementina* v1.0 genome assembly at position 25497528, corresponding to the genetic position of 126.64 cM on the ‘Murcott’ genetic map. This marker was analyzed in 83 citrus genotypes, including 53 polyembryonic, and 30 monoembryonic cultivars. A summary of the results from phenotyping and SNP1 genotyping is indicated in Table 4.

SNP1 marker genotype was in agreement with the mono/polyembryony phenotype in most horticultural groups, except Citrumelo (*C. paradisi × P. trifoliata*), *Fortunella* spp., and *Poncirus* spp. (Table 4 and Supplementary Table S8). For *Fortunella* and *Poncirus* spp., these mismatches are in accordance with the hypothesis reported by Wang et al. [73], who suggest that the parallel evolution of *Fortunella* and *Citrus* has driven the evolution of apomixis in these genera in a differentiated way, resulting in the heterogeneity of genes causing polyembryony in *Citrinae*, a subtribe comprising *Fortunella*, *Poncirus* and *Citrus* genera, among others. Polyembryonic *Poncirus* genotypes do not have MITE insertions in the promoter region of the *CitRWP* gene, and it is also not expressed in nucellar ovule cells, suggesting another causal gene [73,74]. Therefore, the SNP1 marker is fully validated for apomixis characterization of germplasm and hybrids of breeding projects derived from admixture between *C. reticulata* and *C. maxima* or/and *C. medica*, where the polyembryonic trait was inherited from the *C. reticulata* ancestor.

The SNP1 marker will be very useful for the selection of new monoembryonic parents aimed at obtaining new varieties. That is particularly relevant in view of the limited number of monoembryonic female parents available today for the use in breeding programs. In fact, we are routinely using the SNP1 marker in our breeding program to select monoembryonic parents. This, together with the selection for resistance to Alternaria brown spot fungus disease [75], allows us to be more efficient in the selection of parents with improved characteristics. The selected parents are subsequently induced for early flowering by a viral vector based on the *Citrus leaf blotch virus* [76]. This strategy shortens the time needed to recover new improved genetic combinations. In addition, the SNP1 marker will be very useful for the identification of polyembryonic hybrids in rootstock breeding programs which look for polyembryony, because it allows for ease, expense reduction, and consistency of rootstock propagation in the nursery [77]. All these show the high potential of SNP1 for MAS.

## 3. Materials and Methods

### 3.1. Plant Material

A diploid hybrid population, derived from the cross between the diploids ‘Kiyomi’ tangor (IVIA-405) as the female parent and ‘Murcott’ tangor (IVIA-196) as the male parent, was used. The ‘Kiyomi’ tangor is a male sterile and monoembryonic hybrid between the ‘Miyagawa-wase’ satsuma and the ‘Trovita’ sweet orange [78], while the ‘Murcott’ tangor is a male fertile and polyembryonic genotype presumed F1 hybrid of sweet orange and an unknown mandarin. Both parents, belong to the Citrus Germplasm Bank of the Instituto Valenciano de Investigaciones Agrarias (IVIA), located in Moncada, Valencia (Spain). Sixty-two hybrids were recovered and all of them were analyzed by flow cytometry, according to Aleza et al. [79]. Sixty-one were diploids and one triploid hybrid was obtained from female unreduced gamete, which was not included in the genetic analysis. The progeny was grafted in June 2011 onto *C. macrophylla* Wester rootstock at the IVIA experimental orchard for genetic analysis and further studies related with fruit quality.

### 3.2. Plant Genotyping

A total of 61 diploid hybrids from the ‘Kiyomi’ × ‘Murcott’ cross and the two parents were subjected to genotyping by sequencing (GBS), as described by Ollitrault et al. [38]. Genomic DNA was isolated using the Plant DNAeasy^®^ kit (Qiagen, Hilden, Germany), according to the manufacturer’s instructions. The concentration of genomic DNA was adjusted to 20 ng/μL, and the ApekI GBS libraries were prepared following the protocol described by Elshire et al. [30]. The DNA of each sample (200 ng) was digested with the ApekI enzyme (New England Biolabs, Hitchin, UK). Digestion took place at 75 °C for 2 h, and then at 65 °C for 20 min to inactivate the enzyme. The ligation reaction was completed in the same plate as the digestion, again, using the T4 DNA ligase enzyme (New England Biolabs, Hitchin, UK) at 22 °C for 1 h; the ligase was inactivated prior to pooling the samples by holding it at 65 °C for 20 min. For each library, ligated samples were pooled (i.e., 2 multiplex libraries of 96 samples) and PCR-amplified in a single tube. Complexity was further reduced using PCR primers with one selective base (A), as described by Sonah et al. [80]. Single-end sequencing was performed on a single lane of an Illumina HiSeq4000. Keygene N.V. (Keygene, Wageningen, The Netherlands) owns the patents and patent applications protecting its sequence-based genotyping technologies. SNP genotype calling was performed using data from the DNA sequence reads with the TASSEL 5 GBS v2 pipeline [31] (available at https://bitbucket.org/tasseladmin/tassel-5-source/wiki/Tassel5GBSv2Pipeline (accessed on 7 June 2022) with default parameters, to identify good quality, unique, sequence reads with barcodes. These sequences were aligned on the *C. clementina* 1.0 reference genome (available at https://phytozome.jgi.doe.gov, accessed 7 June 2022) using Bowtie v2/2.3.2. For genotype calling, positions with less than five reads were considered as missing data. Next, polymorphic positions were filtered for diallelic SNPs and minor allele frequencies (MAF) over 0.05.

### 3.3. Linkage Analysis and Genetic Mapping

The two-way pseudo-testcross mapping strategy implemented for genetic mapping from progenies, resulting from crosses between two heterozygous parents [81] and used in previous high-density mapping studies in citrus [35,37,38,39,41], was applied to establish the ‘Kiyomi’ and ‘Murcott’ genetic maps. For each map, SNP markers were selected according to their respective heterozygosity for the mapped parent and homozygosity for the other one. Each set of data for the 61 hybrids was filtered to retain markers and hybrids with less than 15% of missing data. Linkage analysis and genetic mapping were then performed using JoinMap5 (https://www.kyazma.nl/index.php/JoinMap/; accessed 7 June 2022). Linkage mapping was performed in the «Hap» option for both ‘Kiyomi’ and ‘Murcott’ tangors. Markers were grouped using the independence LOD score. The phases (coupling and repulsion) of the linked marker loci were automatically detected by the software. Map distances were estimated in cM, using the regression mapping algorithm. After a first mapping round, singletons, i.e., an individual genotype that suggested recombination with its two flanking markers, were identified and replaced by missing data, as recommended by van Os et al. [82] for high density genetic maps. At the same time, a number of individuals displaying an aberrant number of recombination, set by examining the global recombination distribution, were removed, as we considered their genotype calling quality to be insufficient. The synteny and collinearity of both the ‘Kiyomi’ and ‘Murcott’ genetic maps, with the reference clementine genome, were visualized using Circos v0.69-9 [83]; (http://circos.ca; accessed on 7 June 2022) in Galaxy [84]. Marey maps were drawn using Microsoft^®^ Excel^®^ 365 MSO (16.0.15601.20526) to visualize changes in the recombination rate along the genome.

### 3.4. Histological Observations

The male sterility phenotyping was based on the number of pollen grains per anther (NPGA) and the apparent pollen fertility (APF) of hybrids which flowered on each blossom. For this, three flowers per genotype were collected on the day of anthesis. A total of 10 anthers per flower were removed with forceps and placed into 3 different 1.5 mL Eppendorf tubes, with 3 tubes per genotype. Following this, opened Eppendorf tubes were left in a desiccator with silica gel at room temperature, until dehiscence. Dehiscent anthers were confirmed under stereomicroscope, and samples were sorted into three levels, according to pollen grain quantity visually observed: high (Figure 4a–c), moderate-to-low (Figure 4d–f), and very low-to-null (Figure 4g–i). Phenotyping was performed during three flowering periods belonging to the following years: 2019, 2020, and 2021.

Samples were stored at −20 °C until quantification. For pollen grain suspension a staining solution [85], based on Alexander staining [86], was added into the 1.5 mL Eppendorf tube containing the dehiscent anthers. The volume of staining added depended on scored visual observations of dehiscent anthers: 25 μL was added to those samples scored as having null-to-very low quantity of pollen grains, 50 μL to low and moderate samples, and 100 μL to samples scored as moderate-to-high. Eppendorf tubes containing the dehiscent anthers with the staining solution were placed at 70 °C for 30 min. Next, a spin of one hour at 10,000 rpm was performed to separate pollen grains from the theca.

**Figure 5:**
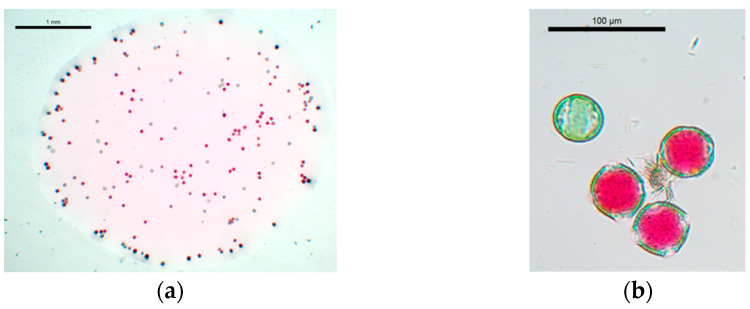
(**a**) Example of a 0.3 μL drop with pollen grains dispersed in the staining solution. (**b**) Detail of viable pollen grains stained magenta-red, and non-viable pollen grains stained blue-green.

The pollen grain dispersion was shaken with a vortex and, immediately, 15 drops of 0.3 μL were placed onto a slide. Drops were photographed (Figure 5a) with a Leica DMLS microscope, and the number of pollen grains per drop was counted with the ImageJ 2.0.0-rc-61/1.52n software [87].

The number of pollen grains per anther (NPGA) was calculated as follows:

(1) $$\mathrm{NPGA}=\left(\mathrm{sum}\;\mathrm{of}\;\mathrm{NPG}/\left(15\times 0.3\right)\right)\times \left(\mathrm{Vol}/10\right)$$

where the sum of NPGA is the total number of pollen grains counted in the 15 drops of 0.3 μL; 10 is the number of anthers; and Vol is the volume (25, 50 o 100 μL) of staining solution added for pollen grain dispersion.

Stain solution colored non-viable pollen grains as blue-green, and viable pollen grains as magenta-red. Since staining solution was used as a liquid medium to disperse the pollen grains, APF and NPGA values were evaluated simultaneously (Figure 5). To determine if there were significant differences between NPGA and APF variables of phenotyped hybrids, a one-way ANOVA test was performed using the Statgraphics Centurion XVI statistical software package, v16.1.03. A *p*-value of less than 0.05 was classified as statistically significant between the averages of NPGA and APF of phenotyped hybrids.

### 3.5. Seed Phenotyping for Polyembryony

Fruits from the hybrids between ‘Kiyomi’ and ‘Murcott’ tangors were harvested when ripe, and seeds were then extracted. Each seed was peeled, eliminating the outer and inner seed coats with forceps. Seeds with only one embryo were classified as monoembryonic, whereas seeds with more than one embryo were recorded as polyembryonic.

### 3.6. Genotype–Phenotype Association

TASSEL v5.2.87 [88] was used to perform the genotype–phenotype association study. Genotype and phenotype data sets were joined by the union of taxa using the Union Join command. Then, the genotype–phenotype association was evaluated using the GLM (general linear model) procedure under the default settings, and the results were displayed using the Manhattan plot graph.

### 3.7. SNP Genotyping

SNP markers were genotyped using KASPar^TM^ technology by KBioscience^®^ (https://www.biosearchtech.com/ (accessed on 7 June 2022). KASPar^TM^ technology uses allele-specific amplification, followed by fluorescence detection. Sample DNA is amplified with a thermal cycler using allele-specific primers based on the SNP locus-flanking sequence (approx. 50 nucleotides on each side of the SNP). The KASPar^TM^ system uses two Förster resonance energy transfer (FRET) cassettes, where fluorometric dye is conjugated to the primer, but quenched via resonance energy transfer when the FRET cassette primer is hybridized with DNA [89].

## 4. Conclusions

GBS was used to genotype 61 diploid hybrids from an F1 progeny recovered from crossing the male sterile and monoembryonic ‘Kiyomi’ tangor as the female parent with the male fertile and polyembryonic ‘Murcott’ tangor as the male parent. Raw sequences were aligned to the clementine genome and 6444 SNPs were obtained. After filtering for SNPs within genes and heterozygous for only one of the parents, we established the genetic map for each parent with the JoinMap.5 software. The two maps, respectively, include 1374 and 697 markers, and encompass 1416.287 and 1339.735 cM for ‘Kiyomi’ and ‘Murcott’. The two maps were globally highly syntenic and colinear with the *C. clementina* v1.0 assembly; however, they confirmed previous constatations for probable small incongruences of the *C. clementina* genome assembly in chromosomes 3, 5, and 9. The progenies were phenotyped for male sterility based on the number of pollen grains per anther (NPG) and apparent pollen fertility (APF) values, as well as for polyembryony. The genotype–trait association study, using the general linear model (GLM), identified a genomic region on linkage group 8 significantly associated with NPGA; however, no association was observed for APF, indicating that NPGA is the major factor for male sterility in the progeny derived from ‘Kiyomi’ × ‘Murcott’. We also identified a genomic region on linkage group 1 significantly associated with polyembryony. The analysis of gene annotation in the region of chromosome 8 associated with NPGA revealed 19 candidate genes implied in pollen development in other plant species. An SNP marker (S08_6142645) based on KASPar technology was developed in the Ciclev10028670m.g gene, appertaining to the papain cysteine protease family, well known for its importance in pollen development. It was validated on a family of uncontrolled hybrids of ‘Kiyomi’ mother plants. We also developed an SNP marker for polyembryony, choosing the SNP in the ‘Murcott’ genetic map closest to the CitRWP gene involved in mandarin apomixis. This marker was fully validated on a collection of varieties derived from *C. reticulata*, *C. maxima,* and *C. medica* ancestors. However, it was not efficient for polyembryonic accessions derived from *P. trifoliata*, and the *Fortunella* sp. This last result is in agreement with previous hypotheses for multiple origins of polyembryony in the true citrus genera.

Male sterility is a desirable trait for seedless breeding and polyembryony is a crucial reproductive feature to be considered in breeding, for both rootstocks and varieties. Marker-assisted selection (MAS) is key in breeding programs, particularly in tree species with long juvenile period, such as citrus, since the selection of target genotypes can be carried out at the seedling stage. In recent years, molecular tagging techniques have evolved, and SNP markers have emerged as an indispensable tool in genetic applications and breeding programs. To our knowledge, the SNP1_25497528 and SNP8_6142645 developed here are the first available to be successfully used in MAS for polyembryony and male sterility in a wide range of citrus genotypes, and will be very useful for MAS breeding programs for varieties and rootstocks.

## Figures and Tables

**Table 1:** Summary of ‘Kiyomi’ and ‘Murcott’ tangors mapping data.

	LG1	LG2	LG3	LG4	LG5	LG6	LG7	LG8	LG9	Total
Kiyomi										
No. of SNPs	118	189	324	128	214	53	180	80	88	1374
Size (cM)	96.8	141.3	237.7	112.3	219.4	107.0	152.3	189.7	159.8	1416.3
Murcott										
No. of SNPs	76	40	75	97	57	81	60	168	43	697
Size (cM)	154.5	138.6	231.3	154.6	145.4	133.2	133.9	144.8	103.4	1339.7

LG: Linkage group.

**Table 2:** ANOVA for number of pollen grains per anther and apparent pollen fertility.

	Sum of Squares	Df	Mean Squares	F Ratio	*p*-Value
No. of pollen grains per anther					
Between hybrids	5.16 × 10^8^	52	9.92 × 10^6^	7.6	0
Within hybrids	1.62 × 10^8^	124	1.30 × 10^6^		
Total	6.78 × 10^8^	176			
Apparent pollen fertility					
Between hybrids	3.58060	52	0.0688576	1.18	0.2369
Within hybrids	5.40762	93	0.0581465		
Total	8.98822	145			

Statistical differences for *p* < 0.05; Df: degrees of freedom.

**Table 4:** Horticultural groups with the number of accessions analyzed with the S01_25497528 SNP marker.

Horticultural Group	Number of Accessions	A:A	A:G	G:G	% Correctly Assigned
Satsuma mandarin	2	0	2	0	100
Clementine mandarin	5	5	0	0	100
Mandarin	14	3	9	2	100
Sweet Orange	9	0	9	0	100
Sour Orange	2	0	2	0	100
Grapefruit	3	0	3	0	100
Lemon	3	1	2	0	100
Lime	2	0	2	0	100
Citron	3	3	0	0	100
Pummelo	3	3	0	0	100
Bergamot	1	1	0	0	100
Mandarin hybrid	18	9	9	0	100
Tangor	6	3	3	0	100
Tangelo	4	1	3	0	100
Citrange	1	0	1	0	100
Citrumelo	1	1	0	0	0
Fortunella	4	4	0	0	25
Poncirus	2	2	0	0	0

The SNP1 allele linked with polyembryony is G. GG and AG allelic configurations correspond with polyembryonic genotypes, whereas AA allelic configurations are monoembryonic. Detailed information of genotypes and phenotypes is provided in Supplementary Table S8, and the KASPar plot in Supplementary Figure S3.

## Data Availability

The data presented in this study are available in the article or as Supplementary Materials.

## Supplementary Materials

The following supporting information can be downloaded at: https://www.mdpi.com/article/10.3390/plants12071567/s1, Figure S1: Synteny and collinearity of the ‘Kiyomi’ tangor genetic map with the reference genome of clementine; Figure S2: Synteny and collinearity of the ‘Murcott’ tangor genetic map with the reference genome of clementine; Figure S3: Plots of the allele signals of KASPar analysis for S08_6142645 and S01_25497528 markers; Table S1: Number of filtered, discarded, and used SNPs markers to perform the linkage mapping of ‘Kiyomi’ and ‘Murcott’ tangors; Table S2: Detail of the ‘Kiyomi’ genetic map, including physical position (clementine reference genome), genetic position, and the gene identifier on which the marker is located; Table S3: Detail of the ‘Murcott’ genetic map, including physical position (clementine reference genome), genetic position, and the gene identifier on which the marker is located; Table S4: Phenotypes of pollen traits and polyembryony for ‘Kiyomi’, ‘Murcott’ and the ‘Kiyomi’ × ‘Murcott’ progeny; Table S5: Markers associated with the number of pollen grains per anther (NPGA) and polyembryony (PE) in the ‘Murcott’ gametes map; Table S6: Gene annotations in the assembled sequence of the genomic region of chromosome 8, associated with male sterility. Annotations related to pollen development are indicated in red letters. (https://phytozome-next.jgi.doe.gov (accessed on 2 March 2023)). Number in brackets close to the gene identifier indicates that it contains an SNP marker. (1) S08_6026790; (2) S08_6050573 and (3) S08_6142645; Table S7: Genotypes for S08_6142645 SNP in different commercial mandarin cultivars; Table S8: Phenotypes for polyembryony and genotypes for S01_25497528 SNP (SNP1) in a germplasm collection.
