# Supplementary material for: Genotyping by Sequencing for SNP-Based Linkage Analysis and the Development of KASPar Markers for Male Sterility and Polyembryony in Citrus
Source: Plants (Basel). 2023 Apr 6;12(7):1567. doi: 10.3390/plants12071567 (PMC10096700; doi:10.3390/plants12071567)
Supplement: Supplementary file 1 [file plants-12-01567-s001.zip › plants-2288451-Proof Done-supplementary-figures.pdf]

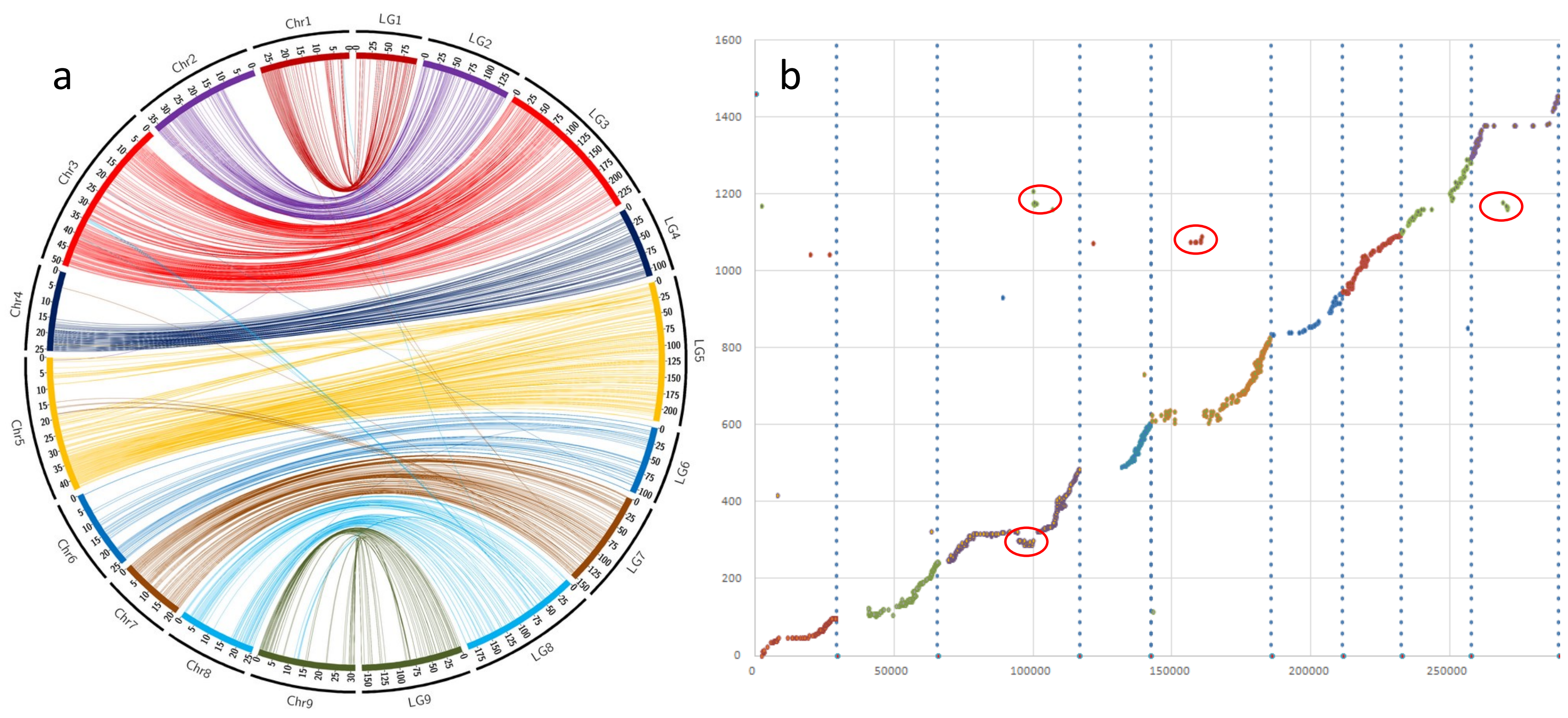

Figure S1. Synteny and collinearity of the 'Kiyomi' tangor genetic map with the reference genome of clementine.

(a) Circos representation of links between the position of markers on the 'Kiyomi' genetic map (LG1 to LG9) and on the chromosome assembly of clementine genome (Ch1 to Ch9). Scales are in cM for LGs and in Mb for chromosomes.

(b) Marey map plot. The x-axis represents the physical positions on the clementine reference genome and y-axis represents the position on the 'Kiyomi' tangor genetic map. The red circles indicates the main incongruences between the two genetic maps and the *C. clementina* genome assembly.

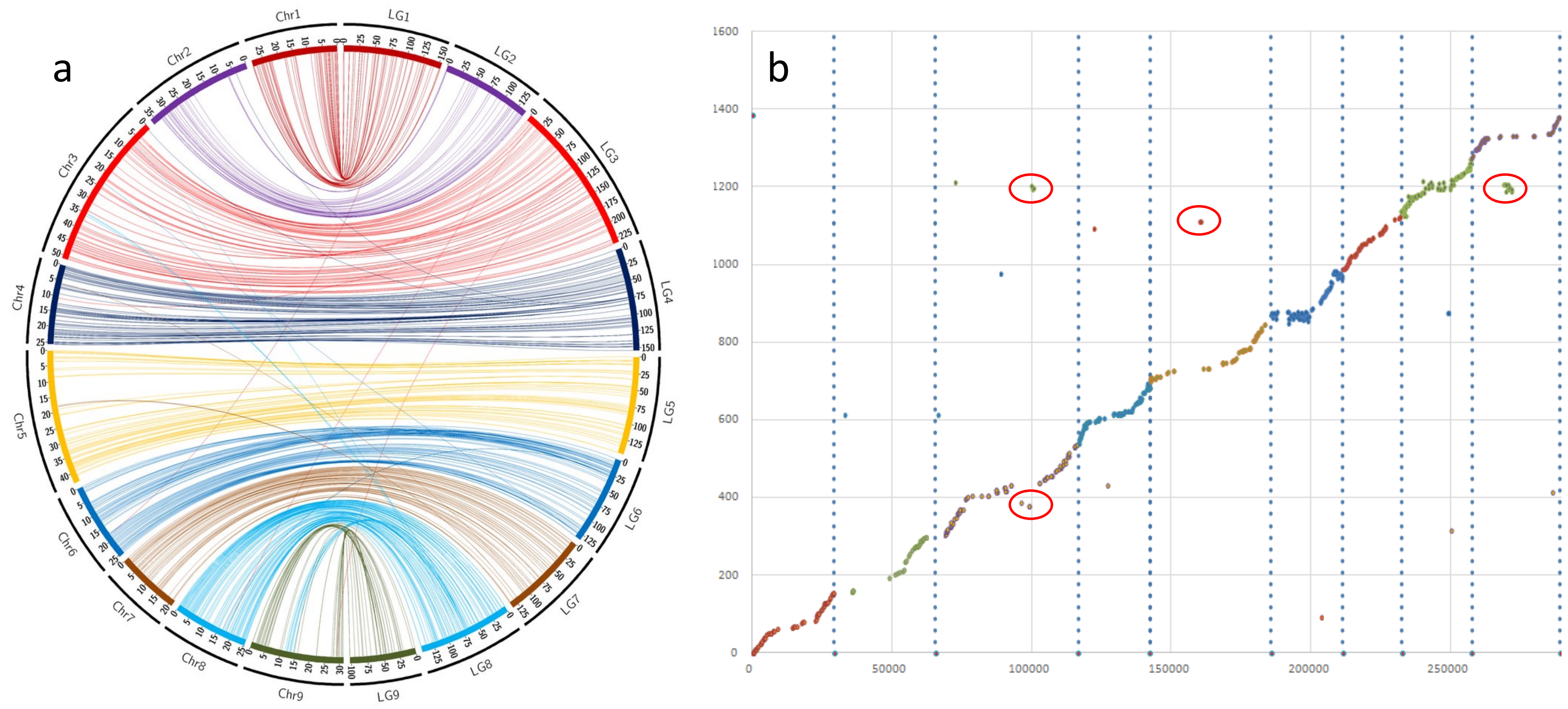

Figure S2. Synteny and collinearity of the ‘Murcott’ tangor genetic map with the reference genome of clementine.
(a) Circos representation of links between the position of markers on the ‘‘Murcott’ genetic map (LG1 to LG9) and on the chromosome assembly of clementine genome (Ch1 to Ch9). Scales are in cM for LGs and in Mb for chromosomes.
(b) Marey map plot. The x-axis represents the physical positions on the clementine reference genome and y-axis represents the position on the ‘Murcott’ tangor genetic map. The red circles indicates the main incongruences between the two genetic maps and the *C. clementina* V1.0 genome assembly.

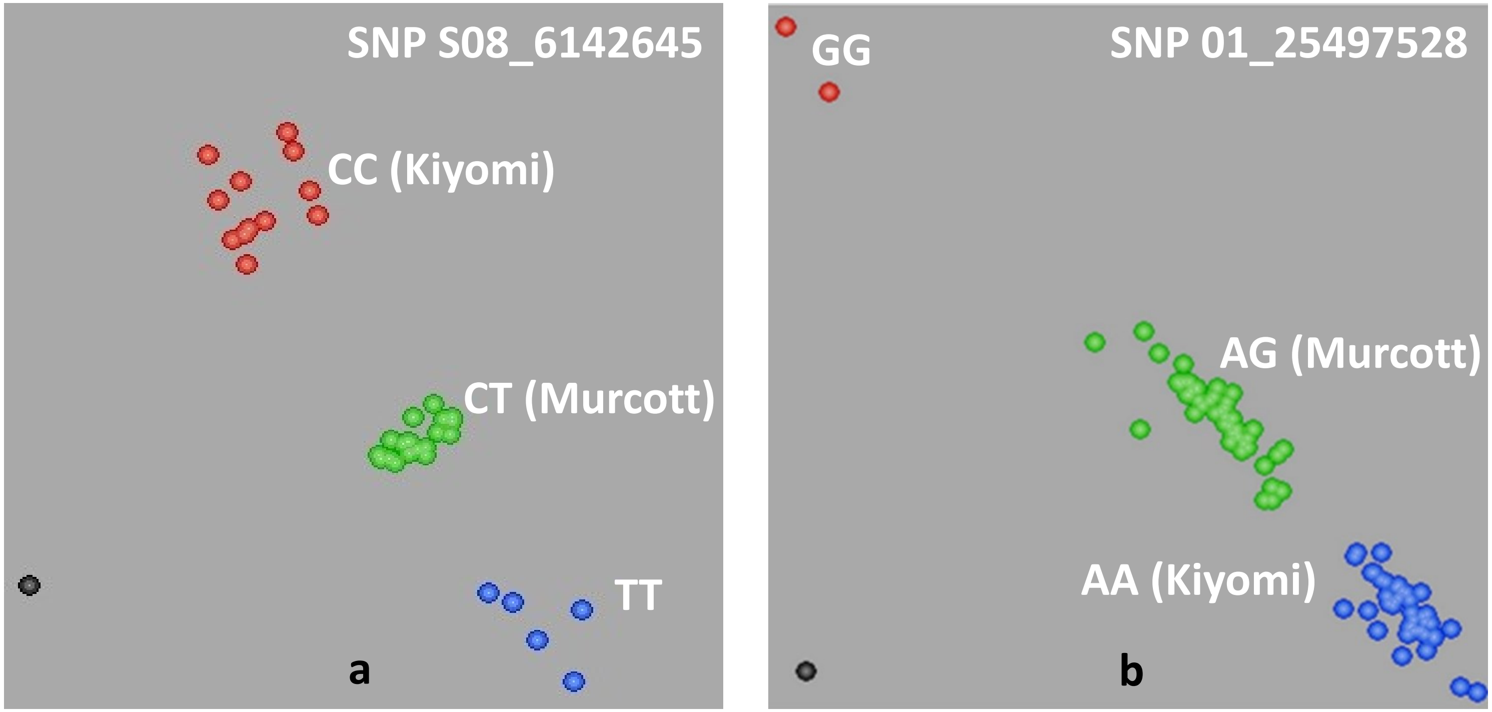

Figure S3. Plots of the allele signals of KASPar analysis for S08_6142645 and S01_25497528 SNP markers.
(a) Plot of the C and T allele signals of the S08_6142645 SNP marker from the cluster analysis of 20 diploid hybrids recovered by the cross-pollination of the 'Kiyomi' tangor with unknown pollen genotype and 11 mandarin varieties.
(b) Plot of the A and G allele signals of the S01_25497528 SNP marker from the cluster analysis of germplasm collection.
